# Comparison of multiple DNA vaccines for protection against cytomegalovirus infection in BALB/c mice

**DOI:** 10.1186/1743-422X-11-104

**Published:** 2014-06-05

**Authors:** Chaoyang Huang, Huadong Wang, Shuting Wu, Haiyan Chang, Lalan Liu, Bo Peng, Fang Fang, Ze Chen

**Affiliations:** 1 College of Life Sciences, Hunan Normal University, Changsha 410081, Hunan, China; 2 Shanghai Institute of Biological Products, Shanghai 200052, China; 3 State Key Laboratory of Virology, Wuhan Institute of Virology, Chinese Academy of Sciences, Wuhan 430071, Hubei, China

**Keywords:** Cytomegalovirus, DNA vaccine, M04 (gp34), M84 (p65), M105 (DNA helicase), IE-1 (pp89), M55 (glycoprotein B)

## Abstract

**Background:**

Human cytomegalovirus (HCMV) causes serious HCMV-related diseases in immunocompromised people. Vaccination is the most effective measure to control infection with the pathogen, yet no vaccine has been licensed till now. We performed a head-to-head comparison of the protective abilities of multiple DNA vaccines in murine model of murine cytomegalovirus (MCMV) infection.

**Methods:**

Five DNA vaccines were constructed. Four encoding MCMV proteins gp34 (m04), p65 (M84), DNA helicase (M105), and immediate-early 1 protein pp89 (IE-1) , respectively, which were reported to induce CD8+ T cell responses, were compared with the one expressing gB (M55), the neutralizing antibody target antigen, for immune protection in BALB/c mice. Mice were immunized with these DNA vaccines 1 to 4 times via intramuscular injection followed by electroporation, and were subsequently infected with a lethal dose (3 × LD50) of highly virulent SG-MCMV. Specific antibodies and IFN-γ secreting splenocytes were detected by immunoblotting and ELISPOT, respectively. Protective abilities in mice provided by the vaccines were evaluated by residual virus titers in organs, survival rate and weight loss.

**Results:**

These DNA vaccines, especially m04, M84 and IE-1, could effectively reduce the virus loads in salivary glands and spleens of mice, but they couldn’t completely clear the residual virus. Survival rates of 100% in mice after a lethal dose of MCMV infection could be reached by more than one dose of M84 vaccine or two doses of m04 or IE-1 vaccine. Immunization with M55 or M105 DNA at four doses offered mice only 62.5% survival rate after the lethal challenge.

**Conclusions:**

The study demonstrated that DNA vaccines could effectively afford mice protection against infection with a highly virulent MCMV and that the protection offered by induced CD8+ T cell immunity might be superior to that by gB-specific antibodies. These results are valuable references for development and application of HCMV vaccines.

## Background

Human cytomegalovirus (HCMV) is a common human herpesvirus, with infection rate of 40 ~ 90% in general population. However, it only causes clinical symptoms in people with a not-fully-developed or compromised immune system, such as newborns, organ transplant patients and people infected with human immunodeficiency virus [1]. Each year, 0.4 ~ 0.7% of newborns have congenital HCMV infections in the United States, of which 12.7% show some symptoms at birth and ultimately develop hepatosplenomegaly, thrombocytopenic purpura, microcephaly, sensorineural hearing loss (SNHL) and/or neurodevelopmental delay [2,3]. No specific treatment is currently available for HCMV infected pregnant women and infants with congenital or perinatal HCMV infections. Vaccination is the most effective measure to control infection with the pathogen. Development of a vaccine against HCMV infection has been identified as a high priority goal in biomedical research [4], yet no vaccine has been licensed till now due to unsatisfactory results of clinical trials on the current HCMV vaccine candidates. Many researches are still in preclinical stage except three, two with a neutralizing antibody inducing gB subunit vaccine plus MF59 adjuvant [5,6] and one with a bivalent DNA vaccine (TransVax™) containing plasmids encoding gB and pp65 formulated with poloxamer CRL1005 and benzalkonium chloride, which have completed phase II clinical trials [7]. The gB/MF59 vaccine exhibited a vaccine efficacy of 50% in CMV-seronegative women and had the potential to decrease incident cases of maternal and congenital CMV infection [6]. The gB/MF59 and the DNA vaccine both could limit the periods of viraemia and consequently the need for antiviral treatment in organ-transplant patients [5,7]. The paper by F. Rieder and C. Steininger reviewed these completed phase II clinical trials on HCMV vaccines [8]. Now evaluation of ASP0113 (formerly TransVax™) is ongoing in phase III trials (NCT01877655 at http://www.clinicaltrials.gov).

A suitable animal model is very useful for the study of HCMV pathogenicity and protective immune responses of HCMV vaccines in humans. However, strict species specificity of CMV has restricted the possibility of establishing an animal model of HCMV infection. The MCMV is similar to HCMV in virion structure, genome organization, gene expression, tissue tropism and latency. Therefore, mouse model of MCMV infection has now been widely used in exploring HCMV infection [9,10]. Similar with HCMV, MCMV is expressed in three sequential phases, immediate early (IE), early (E) or late (L) after infecting host cells, which show coordinate regulation and temporal control [11]. MCMV M123 (IE-1) is the homologous gene of HCMV UL123 (IE-1), and is expressed at the IE phase of MCMV replication as a non-structural protein pp89. M84 is expressed in E phase of replication as a non-structural protein of 65 kD (p65), and its homologous protein pp65 in HCMV is the main antigen for cytotoxic T lymphocyte (CTL) response against HCMV infection in humans [12,13]. The m04 gene is only present in MCMV, and is expressed in E phase of virus replication as gp34 protein and plays an important role in immune escape of MCMV [14,15]. M105 is expressed as DNA helicase of MCMV, which is necessary for MCMV replication in the host. M105 and its HCMV homolog UL105 are highly conserved [16]. M55 gene and its HCMV counterpart UL55 gene both encode the envelope glycoprotein B (gB), the target antigen recognized by CMV-specific neutralizing antibodies. As a new type of vaccine, DNA vaccine could carry antigen-coding genes of a pathogen and induce humoral and/or cellular immune responses *in vivo*. At the same time, DNA vaccine offers a method for selecting effective antigens. Thus we chose to deliver viral components in the form of DNA vaccine, and performed a head to head comparison of five different MCMV DNA vaccines for their capacities in inducing immune responses and providing protection against MCMV infections.

## Results

### Survival rates of mice against a lethal SG-MCMV infection

To evaluate the protection provide by the five DNA vaccines, 420 female, 6 ~ 8 week old BALB/c mice were randomly divided into 21 groups, 20 mice each. One group was the unimmunized control. The other 20 groups were immunized with m04, M84, M105, IE-1 and M55 DNA vaccine at a dosage of 100 μg for 1 ~ 4 times, respectively, via intramuscular injection plus electroporation. The immunization interval was two weeks for multiple doses. Fourteen days after the final immunization, mice were infected via intraperitoneal injection with a lethal dose (3 × LD50) of SG-MCMV. After infection of mice, the survival rate, body weight change and residue virus loads in organs were monitored.

The survival rates of mice were recorded within 21 days post-infection (d.p.i.). As shown in Table 1, mice in control group were unimmunized and all died within 7 days after a lethal virus challenge. Compared with the control, mice immunized with all the plasmids except M55 DNA obtained protection against infection even at one dose of injection, with the survival rates of 81.3%, 75%, 31.3% and 68.8% for m04, M84, M105 and IE-1 groups, respectively. M55 could only provide partial protection (56.3%) for mice at more than 2 doses of injection. Among the four DNA vaccines, i.e. m04, M84, M105 and IE-1,100% survival rate against a lethal MCMV infection was achieved in mice with more than one dose of M84 DNA or more than two doses of either m04 or IE-1 DNA, whereas protective ability provided by M105 was overall poor with multiple immunizations, almost the same as that by M55. The results demonstrated that immunization with m04, M84 or IE-1 DNA vaccine could achieve 100% survival rate against a lethal MCMV infection. Meanwhile, M105, the same as M55 DNA, only provided partial protection.

**Table 1:** **Survival rates of mice after lethal virus challenge**^**§**^

**Vaccine**	**Survival rates (No. of survivors/No. tested)**			
	**One dose**	**Two doses**	**Three doses**	**Four doses**
M04	13/16 (81.3%)^a,b^	15/16 (93.8%)^a,b^	16/16 (100%)^a,b^	16/16 (100%)^a,b^
M84	12/16 (75%)^a,b^	16/16 (100%)^a,b^	16/16 (100%)^a,b^	16/16 (100%)^a,b^
M105	5/16 (31.3%)^a^	6/16 (37.5%)^a^	8/16 (50%)^a^	10/16 (62.5%)^a^
IE-1	11/16 (68.8%)^a,b^	14/16 (87.5%)^a,b^	16/16 (100%)^a,b^	16/16 (100%)^a,b^
M55	0/16 (0%)	0/16 (0%)	9/16 (56.3%)^a^	10/16 (62.5%)^a^
Control	0/16(0%)			

^§^Mice were immunized 1 ~ 4 times, at an interval of 2 weeks, with 100 μg plasmid DNAs. Two weeks after the final immunization, mice were challenged with a lethal dose (3 × LD50) of SG-MCMV. The survival rates of mice were determined 21 days postinfection.

^a^Significant difference compared with control animals, *P* < 0.05.

^b^Significant difference compared with the M55 group at the same immunization frequency.

### Body weight changes of mice after the lethal SG-MCMV challenge

**Figure 1:**
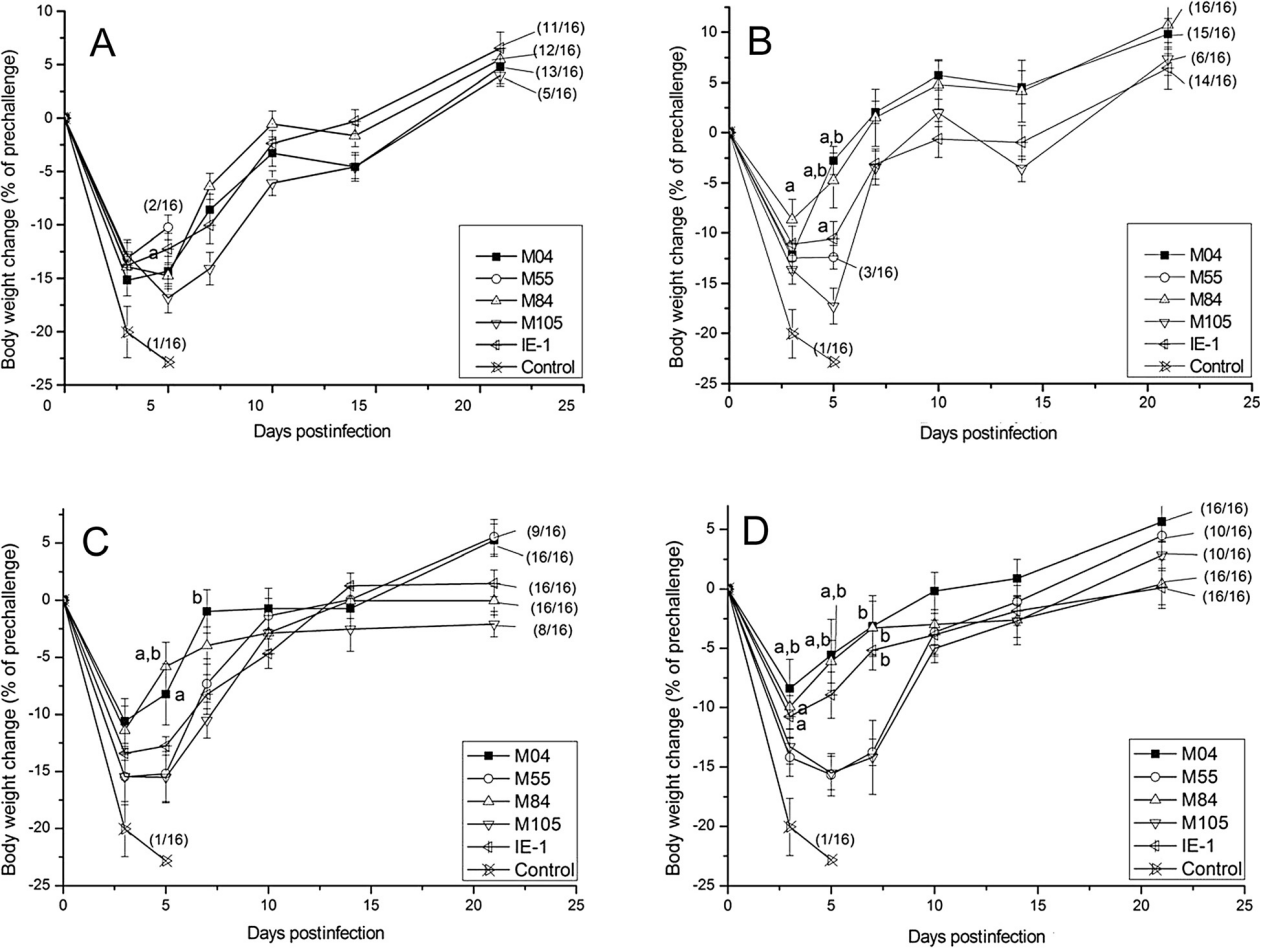
**Bodyweight changes after virus challenge.** Mice were immunized 1 to 4 times **(A-D)** at an interval of 2 weeks, with 100 μg plasmid DNAs encoding M04, M84, M105, IE-1and M55, respectively. Two weeks after the final immunization, mice were challenged with a lethal dose (3 × LD50) of MCMV and the body weights were measured within 3 weeks after the challenge. Data points represent mean ± SD of each group. a. Significant difference compared with control group (*P* < 0.05). b. Significant difference compared with M55 group (*P* < 0.05).

Body weight changes of mice were also recorded within 21 days post-infection. As shown in Figure 1, within the first few days after the challenge, i.e., the acute phase of infection, all mice had drastic weight loss, but significant difference was present in some of the m04, M84 and IE-1 groups compared with the control group and the M55 group. In the control group, the maximal weight loss reached 23% and mice all died within 7 days. The weight losses of all the immunized groups were lower than that of the control group, indicating the protective effects offered by immunization with these DNA vaccines. The maximal weight losses were on day 3–5 p.i., and afterwards mice gradually gained weights. Weight loss moderated along with the increase of the immunization frequency of a DNA vaccine. At the same immunization frequency, the maximal weight losses in m04, M84 and IE-1 groups were lower than those in M55 and M105 groups. By day 21 p.i., all surviving mice had recovered their body weight to pre-challenge levels.

### Residual virus titers in mice organs after challenge

In the process of infection with SG-MCMV, the spleen is a key affected organ, in which the virus load in the infected mice was reported to peak on 5 d.p.i. [17], and salivary gland is another important organ for virus replication, latency and dissemination. We harvested spleens of 4 mice from each group on 5 d.p.i. and salivary glands of another 4 mice from the surviving mice of each group on 21 d.p.i.. The samples were homogenized and analyzed for virus titration by plaque assay on 3T3 cells. The results are shown in Table 2.

**Table 2:** **Virus titers in the spleen and salivary glands of mice after lethal challenge**^**§**^

**Vaccine**	**Spleen virus titers (Log**_**10**_**PFU/ml)**				**Salivary gland virus titers (Log**_**10**_**PFU/ml)**			
	**One dose**	**Two doses**	**Three doses**	**Four doses**	**One dose**	**Two doses**	**Three doses**	**Four doses**
M04	3.9 ± 1.6^a^	3.5 ± 0.2^a,b,e^	3.6 ± 0.2^a^	3.2 ± 0.1^a,b,c,d,e,g^	3.7 ± 0.1	3.7 ± 0.2	3.7 ± 0.2^e^	3.3 ± 0.2^b,c,d,e,g^
M84	3.6 ± 0.2^a^	3.4 ± 0.0^a,e^	3.5 ± 0.1^a,e^	3.3 ± 0.1^a,b,e,g^	3.5 ± 0.2	3.3 ± 0.2^b^	3.2 ± 0.2^b,e^	2.9 ± 0.1^b,c,d,e,f,g,h^
M105	3.8 ± 0.2^a^	3.8 ± 0.1^a^	3.8 ± 0.1^a^	3.6 ± 0.5^a^	4.1 ± 0.1	3.9 ± 0.2^b^	3.9 ± 0.2^b^	3.8 ± 0.4^b^
IE-1	3.6 ± 0.1^a^	3.2 ± 0.4^a,b,e^	2.9 ± 0.2^a,b,e^	2.4 ± 0.3^a,b,c,d,e,g,h^	3.9 ± 0.1	3.7 ± 0.1	3.7 ± 0.1^e^	3.2 ± 0.2^b,c,d,e,g^
M55	3.8 ± 0.1^a^	3.7 ± 0.0^a^	3.7 ± 0.1^a,b,c^	3.5 ± 0.0^c,d,e^	N.D.^j^	N.D.^j^	4.2 ± 0.1	3.9 ± 0.2^d^
Control	5.1 ± 0.1				N.D.^j^			

^§^Mice were immunized 1 ~ 4 times, at an interval of 2 weeks, with 100 μg plasmid DNAs. Two weeks after the final immunization, mice were challenged with a lethal dose (3 × LD50) of MCMV. Spleen virus titers 5 d.p.i. and salivary gland virus titers 3 weeks p.i. were measured. Values represent mean ± SD of 4 mice from each group.

^a^Significant difference compared with the control animals, p < 0.05.

^b^Significant difference compared with the same DNA vaccine at one dose, p < 0.05

^c^Significant difference compared with the same DNA vaccine at 2 doses, p < 0.05.

^d^Significant difference compared with the same DNA vaccine at 3 doses, p < 0.05.

^e^Significant difference compared with the M55 group at the same immunization frequency, p < 0.05.

^f^Significant difference compared with the IE-1 group at 4 doses, p < 0.05.

^g^Significant difference compared with the M105 group at 4 doses, p < 0.05.

^h^Significant difference compared with the m04 group at 4 doses, p < 0.05.

^i^Significant difference compared with the M84 group at 4 doses, p < 0.05.

^j^Not detected, due to death of the mice.

On day 5 after virus infection, the virus titer of spleen detected in the control group was 10^5.1^ PFU/ml. The values in all the immunized mice were significantly lower than that in the control group. In mice immunized with IE-1, the virus loads of spleen declined as the immunization frequency increased and the lowest value was 10^2.4^ PFU/ml at 4 doses. In addition, with 4 doses of injection, the IE-1 group had a significant decrease compared with other groups; the M84 and m04 groups had significantly lower virus titers compared with the M55 and M105 groups. There was no significant difference between the m04 and M84 groups, as well as between M55 and M105 groups.

As for the virus titers in salivary glands on 21 d.p.i., there were no detection data for the control and M55 groups at one and two doses, as no mice survived. The highest virus titer in salivary glands was 10^4.2^ PFU/ml when mice were immunized thrice with M55. Compared with the one-dose immunization, the four-dose immunization significantly reduced the virus loads in the salivary glands of mice. In addition, with 4 doses of injection, the M84, m04 and IE-1groups were significantly lower than the M55 group, but there was no significant difference between the M105 and M55 groups.

The above results demonstrated that these DNA vaccines, especially m04, M84 and IE-1, could effectively reduce the virus loads in organs of mice. However, they were still weak in completely clearing the residual virus.

### Humoral immune response of mice to DNA vaccines

**Figure 2:**
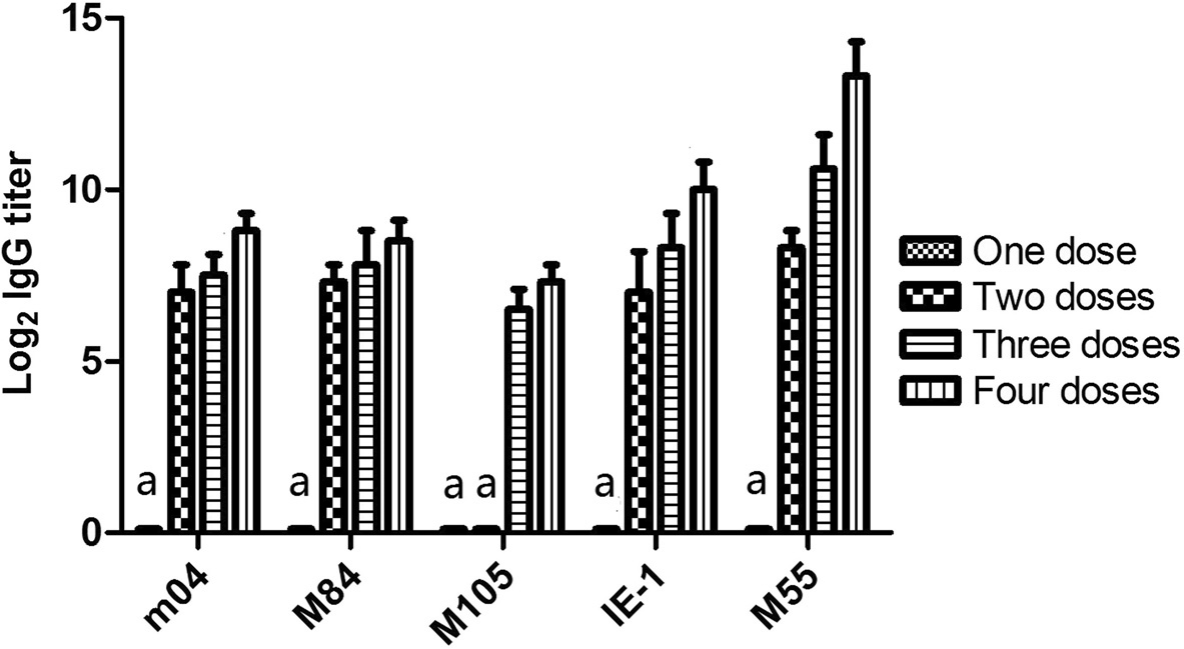
**Detection of antibody responses by immunoblotting.** Mice were immunized 1 ~ 4 times, at an interval of 2 weeks, with 100 μg plasmid DNAs. The serum samples were collected 13 days after the last immunization for titration of antibody responses by immunoblotting. Results are expressed as mean ± SD of 4 mice from each group. a. Not detectable.

Sera samples were collected from 4 mice of each group by tail bleeding 13 days after the last immunization. The specific IgG antibodies to DNA vaccines in sera of mice were analyzed by immunoblotting. The results are shown in Figure 2. All the plasmids could not induce a detectable level of antibody when they were injected only once. When they were immunized twice, all the specific antibody levels were raised except that to M105 antigen. The antibody titers increased along with the increase of the immunization frequency.

### Cellular immune responses of mice to MCMV DNA vaccines

The cellular immune responses were detected by ELISPOT assay in mice immunized four times with m04, M84, M105 and IE-1 DNAs. The plasmid M55 was not involved in this experiment as the H-2^d^ restricted epitope for gB has not been reported anywhere. Additional twenty female mice, 6 ~ 8 weeks old, were equally grouped into 5 groups, 4 mice each. One group was used as control and remained unimmunized. The rest 4 groups were immunized four times with m04, M84, M105 and IE-1 DNAs at a dosage of 100 μg, respectively, followed by electroporation. Two weeks after the last immunization, the splenocytes were separated from the mice, diluted appropriately, and stimulated by addition of the corresponding epitope peptide. IFN-γ secreting splenocytes were detected by ELISPOT assays.

**Figure 3:**
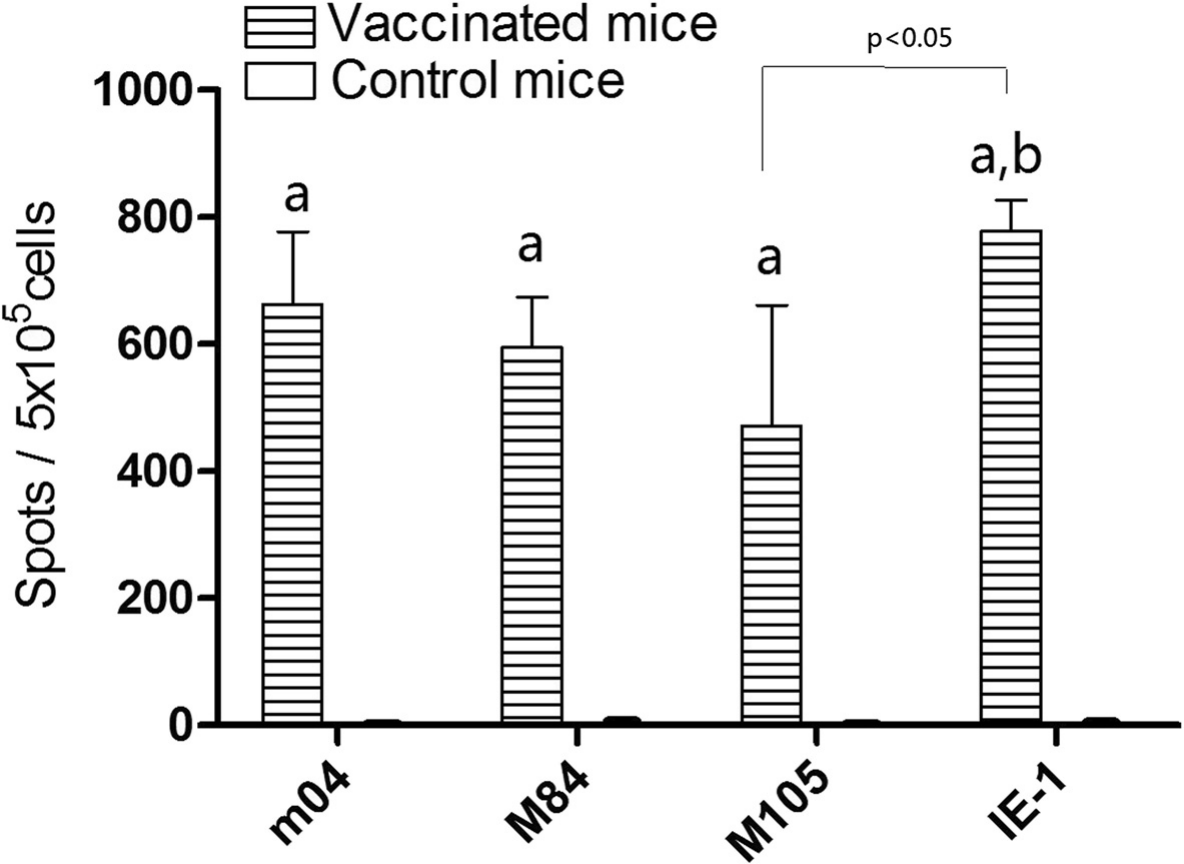
**Detection of IFN-γ secreting splenocytes by ELISPOT assays.** The splenocytes from m04-, M84-, M105- and IE-1- vaccinated mice and control mice were stimulated with 4 μg/ml of the corresponding peptide, respectively. Data shown represent the mean ± SD of each group. a. Significant difference compared with control group (*P* < 0.05). b. Significant difference compared with M105 group (*P* < 0.05).

The ELISPOT results are shown in Figure 3 and demonstrated that m04-, M84-, M105- and IE-1-specific IFN-γ secreting splenocytes could be detected in the corresponding DNA immunization groups. The spot numbers were largely consistent with protection. For every 5 × 10^5^ splenocytes, the number of induced IFN-γ secreting splenocytes was highest in the IE-1 DNA vaccine group, reaching 779 ± 49.0 spots (range, 703 to 831). The m04 group came next and reached 662 ± 114.2 spots (range, 474 to 681). The M84 group had 595 ± 78.6 spots. The M105 group, which showed relatively weak protection, had 472 ± 190 spots. In contrast, only less than 10 spots were detected when the epitope peptides were used to stimulate splenocytes from the unimmunized mice. The IFN-γ secreting splenocytes in each of the immunized groups were significantly (*p* < 0.05) higher than those in the control group. Among the four immunized groups, the IE-1 group had a significantly higher number of spot than the M105, and there was no significant difference between other groups.

## Discussion

Because of strict species specificity of CMV, it is hard to establish an animal model of HCMV infection. The mouse model of MCMV infection has therefore been generally used in development of HCMV vaccines. As we know, CMV-specific humoral immunity and CMI both play critical roles in controlling CMV infection. Neutralizing antibodies could effectively decrease primary infection of CMV, and also block CMV infection of the fetus [18-20]. However, replicating or latent viruses in the host are cleared mainly by CMI. In transplant recipients with impaired CMI, HCMV specific T-lymphocyte responses and HCMV diseases had inverse correlation [16]. CD8^+^ T cells responded to a large panel of HCMV antigens expressing during different phases of replication and played an important role in the overall pathogenesis of HCMV associated diseases [21]. DNA vaccine offers a method for selecting effective antigens. Among the five MCMV DNA plasmids constructed in our present study, M55 DNA that codes for the surface glycoprotein gB induces the neutralizing antibody, while the rest four have been reported to induce CD8+ T cell responses [16,22,23]. M55 DNA could only provide partial protection even at four doses. The protections for mice offered by m04, M84 or IE-1 plasmid were proved to be superior to that by M55. Immunization with m04, M84 or IE-1 plasmid at more than one or two doses could provide 100% survival rate for mice against the lethal challenge. A series of MCMV DNA vaccines, including M105, IE-1, M84 and m04, had been evaluated for their protective abilities in separate papers [16,22-24], and proved that they could afford mice some degree of resistance against a sublethal SG-MCMV infection. However, in the present paper, we made a head-to-head comparison of these MCMV DNA vaccines in the same animal model and we think that challenge of mice with a lethal dose of the virus would gain a more direct and intuitional result than a sublethal challenge.

Besides the survival rate, the residue virus titers in organs and the body weight changes of mice are also very important factors in assessing the CMV vaccines. MCMV infects mice via i.p. and acutely replicates in multiple organs, including spleen, liver, lung, and salivary glands. Virus peaks in spleen and liver around 5 d.p.i., drops to the limit of detection by 9 d.p.i. [25]. Meanwhile, the spleen is the most important organ for T cell responses. We tested the virus titers of spleen in mice 5 d.p.i. (Table 2). Compared with the control, all the vaccinated groups showed the significantly lower virus titers of spleen. The lowest titer appeared in the IE-1 group at 4 doses, and the titers in the m04 and M84 groups were similar, a little higher than that in the IE-1 group. The highest virus titer in spleens of mice after 4 doses of immunization was the M105 group. This result corresponded with that of the IFN-γ secreting splenocytes by ELISPOT assay, in which IE-1 group had the highest number of IFN-secreting splenocytes (Figure 3). In the study performed by Ye *et al*. [16,23,24], immunization of IE-1 plasmid also showed a relatively lower virus titer of spleen. The reason might partly lie in the immunodominance status of the antigen epitopes. In BALB/c mice, the *H-2*^*d*^ restricted epitope (168-YPHFMPTNL-179) in the IE-1 protein is immunodominant [26] and those in the M84 and m04 proteins are subdominant, whereas in the M105 protein is intermediate [27]. Even so, studies by both Spector group [16,22,23] and Holtappels group [28] had come to a conclusion that, in BALB/c mice, the protective potential of an antigen was independent of the immunodominance status of its epitope [27]. Apart from the spleens of mice, we also tested the salivary glands, which are the main organ for CMV replication and latency [25], for their virus titers. The residue virus loads of salivary glands in the m04, M84 and IE-1 groups at 21d.p.i. were all lower or significantly lower than those in the M55 and M105 groups at the same immunization dose (Table 2). In addition, the body weight of mice varied obviously and accordingly during the first days after the lethal dose infection (Figure 1). The mice lost less weight when a plasmid provided better protection. Thus the survival rate, organ virus titers and body weight changes could be considered together to evaluate the protective abilities of the CMV vaccines in such a murine model of lethal-dose virus infection.

Administration of the gB gene by homologous DNA prime-boost may not be the most efficient delivery system to develop neutralizing antibodies, yet the gB subunit vaccine adjuvanted with MF59 only exhibited a vaccine efficacy of 50% in CMV-seronegative women [6]. Therefore, in this study, we explored the importance of the cellular immune responses against CMV infection. We compared the protective potential of DNA vaccines expressing a single MCMV antigen and demonstrated that the antigens such as M84, IE-1 and m04, which are the specific CD8^+^ T cell response inducer, protected mice with 100% survival rate and with obviously reduced virus loads in organs. However, they were still weak in completely clearing the residual virus. One reason for this may lie in the biological nature of latent infection of CMV, and another may be that only a single antigen was involved in the immune responses. A study by Elkington et al. demonstrated that in healthy immune individuals, CD8^+^ T cell responses were directed towards multiple antigens (pp65, pp50, pp28, pp150, IE-1, US2, US3, US6, US11, UL16, UL18, gB, and gH), which may contribute to controlling HCMV replication, and that more than 40% of the T-cell reactivity was directed towards antigens other than pp65 and IE-1 [21]. Thus, a multi-component vaccine should be developed in order to provide a more satisfied protection against CMV infection. These studies are currently in progress.

## Conclusions

The results presented in this study demonstrated that the m04, M84 or IE-1 DNA provided more effective protection than M55 or M105 DNA for mice against a lethal virus challenge. It means that the protection offered by induced CD8+ T cell immunity might be superior to that by gB-specific antibodies. These results are valuable references for development and application of HCMV vaccines.

## Methods

### Mice, cell, and virus

Specific-pathogen-free (SPF) female BALB/c mice (*H-2*^*d*^), aged 6–8 weeks, were purchased from the Center for Disease Control and Prevention in Hubei Province, China, and were maintained in SPF conditions prior to infection.

MCMV Smith strain was propagated in NIH 3T3 cells (ATCC CRL 1658). The virus obtained from the cell culture was referred to as TC-MCMV (tissue culture-derived MCMV). TC-MCMV was propagated and purified by ultracentrifugation as described by Brune et al. [29]. The virus, which was *in vivo* passaged for virulence enhancement and isolated from salivary glands of the infected mice, was referred to as SG-MCMV (salivary gland-derived MCMV). The high-virulence SG-MCMV stock was prepared through 14 times of *in vivo* passages and was used in challenge experiments. Challenge was performed with 3× LD50 virus stock.

### Plasmid DNAs and peptide

Plasmids pcDNA3.1/m04, pcDNA3.1/M84, pcDNA3.1/M105, pcDNA3.1/IE-1 and pcDNA3.1/M55 were constructed by cloning the PCR products of m04, M84, M105, IE-1and M55 gene from the MCMV smith strain into the plasmid expression vector pcDNA3.1/myc-His B (Invitrogen, CA), which encoded gp34, p65, DNA helicase, pp89 and glycoprotein B (gB) proteins, respectively. PCR amplifications for m04, M84, M105 and M55 genes were carried out using the following paired sense and antisense primers: 5′AGaagcttATGTCTCTCGTATGTCGGC3′ (containing Hind III site) and 5′GCctcgagGGTTAGTTACTCTTAAGCGGT3′ (containing Xho I site) for m04, 5′GCaagcttCATGTCGGTCAACGTTTACT3′ (containing Hind III site) and 5′GCtctagaGGCTCTGTCTGTTTGTCTATG3′ (containing Xba I site) for M84, 5′GCgaattcGTTGATCATGGAGAAGAG3′ (containing EcoR I site) and 5′GCtctagaGTCAGAAAACCAGAGTG3′ (containing Xba I site) for M105, 5′GTaagcttGATCGCTGAACAACGCTC3′ (containing Hind III site) and 5′GAggatccTCCTCGCAGCGTCTCCAAT3′ (containing BamH I site) for M55. As for IE-1, its ORF has a four-exon structure, in which three exons encoded pp89 protein [11]. We had to construct the continuous IE-1 gene by overlap-PCR with the three pairs of primers: 5′TAggatccGAGATGGAGCCCGCCGCAC3′ (containing BamH I site) as IE-1 sense, 5′GGCGACATGAGCTGGCACCTTGTCTGATGGGTAGAC3′ as Exon 2 antisense, 5′GTGCCAGCTCATGTCGCC3′ as Exon 3 sense, 5′ACAACAGAACGCTCCTCACTGCAGCATGCTTGATGG3′ as Exon 3 antisense, 5′GAGGAGCGTTCTGTTGTC3′ as Exon 4 sense, and 5′CGgaattcGGGCTTGTGGATTCACTTCT3′ (containing EcoR I site) as IE-1 antisense. All the plasmids were propagated in E. coli XL1-blue bacteria and purified using NucleoBond Xtra Maxi purification kits (Macherey-Nagel, Germany).

As the H-2^d^ restricted epitope for gB has not been reported anywhere thus far, we only obtained epitope peptides of the other four MCMV proteins. The peptide 243-YGPSLYRRF-251 for gp34 protein [30], 297-AYAGLFTPL-305 for p65 protein [31], 207-TYWPVVSDI-215 for DNA helicase [28], and 168-YPHFMPTNL-176 for pp89 protein [13] were synthesized by Shanghai Sangon Biological Engineering Technology & Services Co., Ltd., and were used for IFN-γ ELISPOT assay.

### Immunization

*In vivo* electroporation was carried out according to the method described by Aihara and Miyazaki [32]. Mice were immunized with plasmid DNA dissolved in 100 μl of Tris-EDTA buffer at a dosage of 100 μg by injection into the left and right quadriceps muscles, 50 μg each. After the injection, a pair of electrode needles with 5 mm apart was inserted into the muscle to cover the DNA injection sites and electric pulses were delivered using an electric pulse generator (Electro Square Porator T830 M; BTX, San Diego, CA). Three pulses of 100 V each, followed by three pulses of the opposite polarity, were delivered to each injection site at a rate of one pulse per second. Each pulse lasted for 50 ms. The non-immunized mice were set up as controls. Mice were immunized 1 ~ 4 times, at an interval of 2 weeks.

### Specific antibody assay

Serum samples of mice were collected 13 days after each immunization and stored at −20°C. Titers of IgG Abs against the respective viral proteins were measured by using immunoblotting as previously described [33]. Confluent 3T3 cells were infected with MCMV (m.o.i = 1) for 1.5 hours at 37°C. Unadsorbed virions were removed and infection medium was added. Cells were collected 18 hours and three days postinfection, respectively, and then lysed. Cell lysates were separated using SDS-PAGE (10% gel), blotted onto nitrocellulose membranes. The membranes were blocked with the non-fat milk, dried, and cut into longitudinal slips of 2 mm width. Serial twofold dilutions of sera from each group of immunized mice were prepared and each diluted serum sample was used to incubate each membrane slip. Antibody binding was detected using HRP-labeled goat anti-mouse IgG (a gift from Dr. Rushi Liu) and HRP-DAB detection kit (Sangon, China). The highest serum dilution, giving a positive staining of slip at the site corresponding to the molecular weight of each viral protein, represented the immunoblotting Ab titer of each molecule.

### IFN-γ ELISPOT assay

Fourteen days after the 4th immunization, four mice in each group (including M04, M84, M105, and IE-1 DNA groups as well as the control) were euthanized for isolating spleen cells. Secretion of IFN-γ was detected by ELISPOT using precoat ELISPOT kit (DAKEWEI, China). According to the instruction manual, 96-well PVDF plates (Millipore, Bedford, MA) were coated with 100 μl of 10 μg/ml rat anti-mouse IFN-γ Ab in PBS and incubated at 4°C overnight. Next, 5 × 10^5^ lymphocytes isolated from the spleen cells were added to the wells in triplicate, stimulated with 4 μg /ml of the corresponding synthesized peptide, and incubated at 37°C for 20 h. The lymphocytes were then removed, and 100 μl of biotinylated anti-mouse IFN-γ Ab was added to each well and incubated at 37°C for 1 h. Subsequently, 100 μl of properly diluted Streptavidin-HRP conjugate solution was added and incubated at room temperature for 1 h. Finally, the plates were treated with 100 μl of AEC substrate solution and incubated at room temperature for 25 min in the dark. The reaction was stopped by washing with demineralized water. The plates were air-dried at room temperature and read using an ELISPOT reader (Bioreader 4000; Bio-sys, Germany). Medium backgrounds were consistently <10 spots per 5 × 10^5^ splenocytes.

### Infection

Fourteen days after the last immunization, mice were challenged with a lethal dose (3 × LD50, 200 μl/mouse) of the high-virulence SG-MCMV by i.p. injection. Body weights and survival rates of mice were recorded within 21 days post-infection.

### Virus titrations

Five days after the virus challenge, four mice from each group were sacrificed and the spleens were taken aseptically for titration of spleen residual virus. On day 21 post-challenge, another 4 mice were taken randomly from the remaining mice for aseptic collection of salivary glands to determine the viral load. Spleens and salivary glands were homogenized in 1:10 (w/v) volume with minimal essential media (MEM) containing 2% calf serum. The homogenized fluids were centrifuged and the supernatants were stored in aliquots at −80°C.

Viral loads were determined using a plaque forming unit assay. Briefly, organ homogenates were 10-fold serially diluted and 100 μl of each dilution was used to infect 3T3 cells cultured in 48-well plates. Infections were performed in triplicate. After 1 hour of absorption, supernatant was sucked away and to each well was added 0.5 ml viscous medium. After incubation for 4 days, viral plaques were counted and the viral PFU per milliliter were calculated. The virus titer in each experimental group was presented as the mean of mice samples in that group ± SD.

### Statistics

In this study, the experiments were repeated 3 times. The survival rates of the mice in the test and control groups were compared by using Fisher’s exact test. Body weight changes between the groups were compared by Student’s t-test. Other results between the groups were compared by ANOVA analysis. If P-value is less than 0.05, the difference was considered significant.

## Abbreviations

MCMV: Murine cytomegalovirus; HCMV: Human cytomegalovirus; SG: Salivary gland; SPF: Specific pathogen free; i.p: Intraperitoneal; i.d: Intradermal; i.m: Intramuscular; CMI: Cell-mediated immunity; CTLs: Cytotoxic T lymphocytes; PFU: Plaque forming unit; d.p.i: Days postinfection; ELISPOT: Enzyme-linked spot assay.

## Competing interests

The authors declare that they have no competing interests.

## Authors’ contributions

CYH did most of the experimental work and drafted the manuscript. HDW, STW, and HYC participated in the analysis of humoral responses and plaque assays. LLL, and BP participated in the immunization of mice. FF participated in its design and coordination. ZC revised the manuscript for important intellectual content and gave final approval of the version to be published. All authors read and approved the final manuscript.
